# Effect of Super-Bond C&B and self-adhesive dual-cured resin cement on the fracture resistance of roots with vertical root fracture

**DOI:** 10.15171/joddd.2019.024

**Published:** 2019-08-14

**Authors:** Ezgi Doğanay Yıldız, Hakan Arslan, Nilay Ayaz, Mustafa Gündoğdu, Alper Özdoğan, Eyup Candas Gundogdu

**Affiliations:** ^1^Department of Endodontics, Faculty of Dentistry, Kırıkkale University, Kırıkkale, Turkey; ^2^Department of Endodontics, Faculty of Dentistry, Health Sciences University, Istanbul, Turkey; ^3^Department of Endodontics, Faculty of Dentistry, Atatürk University, Erzurum, Turkey; ^4^Department of Prosthodontics, Faculty of Dentistry, Atatürk University, Erzurum, Turkey; ^5^Department of Oral and Maxillofacial Surgery, Faculty of Dentistry, Atatürk University, Erzurum, Turkey

**Keywords:** Fracture resistance, self-adhesive dual-cured resin, Super-Bond, vertical root fracture

## Abstract

***Background.*** Vertical root fracture might occur during root canal preparation, obturation, post procedures or endodontic treatment.

***Methods.*** Fifty-four single-rooted human teeth were decoronated to obtain a standardized length. The root canals were enlarged up to #50 and obturated with gutta-percha and root canal sealer. Eighteen teeth were used as a control group, and vertical root fracture was induced in the remaining teeth. The samples were randomly divided into three groups, as follows: control group (without vertical root fracture), Super-Bond C&B group (fragments were attached with Super-Bond C&B), and self-adhesive dual-cured resin cement group (fragments were attached with self-adhesive dual-cured resin cement). Each specimen was subjected to a fracture resistance test, and data were statistically analyzed using chi-squared test, one-way ANOVA and post hoc Tukey tests (P=0.05).

***Results.*** The fracture resistance values of the control and Super-Bond C&B groups were higher than those of the self-adhesive dual-cured resin cement group (P<0.05). However, there were no significant differences between the control and Super-Bond C&B groups (P>0.05).

***Conclusion.*** Within the limitations of the present study, Super-Bond C&B was beneficial in obtaining higher fracture resistance in endodontically treated roots with vertical root fracture.

## Introduction

Vertical root fracture (VRF) is one of the major reasons for extraction of endodontically treated teeth.^1^ VRF has been defined as a longitudinally oriented fracture of the root, extending from the root canal to the periodontium.^2^ It might occur during root canal preparation, obturation, post procedures or endodontic treatment.^3^ Clinical and radiographic findings of the VRF generally involve pain, deep periodontal pocket, sinus tract and J-type radiolucency. Treatment options include extraction, resection of the affected root for multiple-rooted teeth or reattachment of the fragments.^2,4-7^

Dual-cured resin cement,^5,6,8,9^ polyethylene fiber,^6,9^ glass fiber,^6,9^ and Super-Bond C&B (Sun Medical Co, Ltd. Moriyama, Japan)^4,10^ have been used to reattach the fragments. In most of these studies, dual-cured resin cement was used to attach the fragments as a main bonding material.^5,6,8,9^ Super-Bond C&B, a tri-nbutylborane (TBB) initiated adhesive resin, is a self-cured adhesive resin cement based on methyl methacrylate. According to the manufacturer, it exhibits excellent bond strength to dentin. In previous case reports and clinical studies, Super-Bond C&B was found to be an effective agent for intentionally replanted and attached teeth with VRF.^4,10^ However, in the literature there is no in vitro study evaluating the effect of Super-Bond C&B on the fracture resistance of roots with VRF. Therefore, the aim of the present study was to evaluate the effect of Super-Bond C&B and self-adhesive dual-cured resin cement on the fracture resistance of roots with VRF. The null hypothesis was that there are no significant differences in terms of fracture resistance between the groups.

## Methods

Fifty-four freshly extracted single-rooted human teeth with similar dimensions were used for this study. The teeth were extracted for the reasons unrelated to this study. The teeth were immersed in 0.5% chloramine-T solution (Merck, Germany) for 48 hours, and soft tissues and calculi were mechanically removed from the root surfaces using a periodontal scaler. The teeth were inspected by using an optical loope (Opt-on Loupe; Orangedental GmbH & Co. KG, Germany) under ×2.7 magnification to exclude teeth with cracks. Digital radiographs were taken of the extracted teeth, and teeth with more than one canal and calcification were excluded from the study. The crowns of teeth were removed to obtain a standardized root length of 15 mm. Mesiodistal and buccolingual lengths of the teeth were measured using a digital caliper and the teeth were divided into three groups according to the mesiodistal and buccolingual lengths. According to the one-way ANOVA, there were no significant differences between the groups in terms of mesiodistal and buccolingual lengths (P>0.05). The teeth were stored in saline solution until use in the experimental procedures.

The working length was established and the root canals were prepared up to #50 using Reciproc R50 (Reciproc; VDW GmbH, Munich, Germany). The root canals were irrigated with 2 mL of %1 NaOCl after each pecking motion; 5 mL of %1 NaOCl was used for final irrigation. The root canals were dried with paper points and filled with gutta-percha (DiaDent Group International, Bunaby, Canada) and root canal sealer (Sealapex; Kerr Corporation, Orange, CA, United States) using lateral condensation technique. Eighteen samples were served as a control group, and intentional VRFs were induced in the remaining samples. To create VRF, the specimens were placed on the lower plate of an Instron universal testing machine (Instron Corp., Canton, MA, USA) and loaded in a vertical direction at 1 mm/min speed until they fractured vertically. The teeth were divided into three groups as follows:

**Control group:** Teeth with no VRF.

**Super-Bond C&B group:** After induction of VRF, the fragments were attached by using Super-Bond C&B (Sun Medical Co, Ltd. Moriyama, Japan). The bonding procedure was performed according to the manufacturer’s recommendations. The fractured surfaces of the fragments were then etched with dentin-etching acid (Super-Bond C&B; Sun Medical Co, Ltd) for 10 seconds and rinsed with distilled water. After slightly drying the surfaces, Super-Bond C&B was mixed using bulk-mix technique (polymer powder, 4 drops of monomer and 1 drop of catalyst V) and placed between the fragments.

**Self-adhesive dual-cured resin cement group:** After induction of VRF, the fragments were attached by using self-adhesive dual-cured resin cement (VOCO GmbH, Cuxhaven, Germany). The bonding procedure was performed according to the manufacturer’s recommendations. The fractured surfaces of the fragments were rinsed with distilled water and slightly dried. Bifix SE (VOCO GmbH) was placed between the fragments and light-cured for 10 seconds.

After the fragments were attached and bonded, all the specimens were stored in distilled water for one day at 37°C. All the teeth were embedded in a block of self-curing acrylic resin (Vipi Flash; Vipi Industria, Sao Paulo, Brazil), exposing 2 mm of the coronal part. Periodontal ligament simulation was performed using a light-body silicone impression material. The acrylic blocks were placed on the lower plate of an Instron universal testing machine (Instron Corp), and a steel ball was mounted on the testing machine. The force was applied at a constant crosshead speed of 1 mm/min until the sample fractured. The force at fracture in each sample was recorded in Newtons.

The failure type was recorded and classified as favorable (would allow repair) or catastrophic (nonrestorable). The favorable failure type was located at the cervical third, whereas catastrophic failure was located at the middle or apical thirds. Data were first analyzed with Kolmogorov-Smirnov (P=0.693) and Levene's tests (P=0.062) to determine whether they were parametric or non-parametric. Data were then analyzed with one-way ANOVA and post hoc Tukey tests at %95 confidence interval (P=0.05). Data for failure types were analyzed using chi-squared test (P=0.05).

## Results

The mean fracture resistance values for the groups are shown in Table 1. The fracture resistance values of the control and Super-Bond C&B groups were higher than those of the self-adhesive dual-cured resin cement group (P<0.05). However, there was no significant difference between the control and Super-Bond C&B groups in terms of the fracture resistance values (P>0.05).

**Figure 1:**
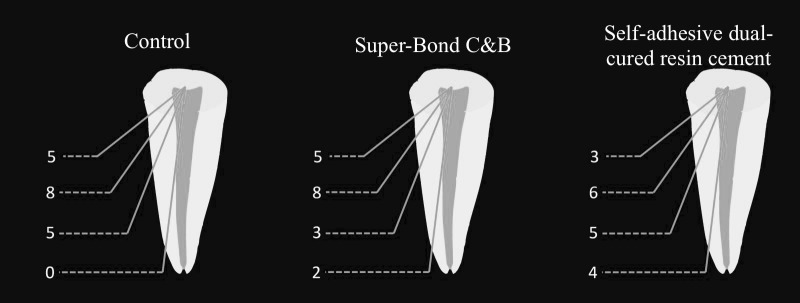
Failure types in terms of the groups. The favorable failure type was located in the cervical third; on the other hand, catastrophic failures were located in the middle or apical thirds.

The modes of failure are listed in Table 1. Favorable failure was the most frequent type of failure in the control and Super-Bond C&B groups (Figure 1). However, there was no significant difference between the groups in terms of the failure type (P>0.05).

**Table 1:** Fracture resistance details of the groups (Newtons)

**Groups**	**No.**	**Mean**	**SD**	**Min**	**Max**	**Failure Type**	
						**Favorable**	**Catastrophic**
**Control group**	18	851.05^a^	270.12	396	1364	13 (72.2%)^a^	5 (27.8)^a^
**Super-Bond C&B**	18	722.05^a^	242.45	300	1224	13 (72.2%)^a^	5 (27.8)^a^
**Self-adhesive dual-cured Resin cement**	18	481.11^b^	151.31	181	763	9 (50%)^a^	9 (50%)^a^

## Discussion

Different letters show statistically significant differences between the groups in the same column (P<0.05).

Most endodontically treated teeth have been reported to be extracted because of VRF after the treatment.^1^ Reattachment of the fragments, one of the treatment options, includes the extraction of the fragments, attachment of the fragments and intentionally replantation of the tooth. For this purpose, dual-cured resin cement^5,6,8,9^ and Super-Bond C&B^4,10^ have been used in previous reports. However, in the literature there is no in vitro study evaluating the effect of Super-Bond C&B on the fracture resistance of roots with VRF. Therefore, the aim of the present study was to evaluate the effect of Super-Bond C&B and self-adhesive dual-cured resin cement on the fracture resistance of roots with VRF. Because there were significant differences between these groups, the null hypothesis was rejected.

The main finding of this study was that reattachment of fragments of VRF with Super-Bond C&B was similar to that of the roots without VRF. Unver et al^4^ reported the treatment of a vertically fractured tooth by intentional replantation after root canal treatment and repair with Super-Bond C&B. Follow-up at the 36-month interval revealed that the tooth was asymptomatic, radiographically sound with reduced deep periodontal pockets and vertical bone loss. The authors reported that intentional replantation after repairing fractured fragments with Super-Bond C&B extraorally is a treatment option. Similarly, Nizam et al^10^ evaluated the clinical outcomes of 21 intentionally replanted maxillary single-rooted teeth with VRFs after being repaired extraorally using Super-Bond C&B. According to the results, adhesive cementation with Super-Bond C&B and intentional replantation were an effective treatment modality for this group of vertically fractured maxillary single-rooted teeth. Our results confirm the results of previous case reports.

In the majority of previous case reports, dual-cured resin cements were used to attach the fragments of teeth with VRF. Ozturk and Unal^5^ reported successful treatment of a vertically fractured tooth which was reconstructed with a self-etching dual-cured adhesive resin cement and intentionally replanted. At a follow-up consultation 4 years later, the tooth was asymptomatic and attachment gain and bone regeneration were observed. In another case report by Moradi Majd et al,^8^ the fragments of the tooth with VRF were extracted and the fracture line was treated with adhesive resin cement. Follow-up at 12 months revealed that the tooth was asymptomatic. However, in these studies, the dual-cured resin cement was not compared with any control group. As for in vitro studies in which dual-cured resin cement was compared with control groups, Kumar et al^9^ evaluated the resistance to fracture of vertically fractured and reattached fragments bonded with fiber-reinforced composites, and found that vertically fractured teeth can be treated by obturating the root canal space with dual-cured adhesive resin cement or by adding polyethylene fiber or glass fiber to increase fracture resistance of the reattached tooth fragments. Similarly, Sen et al^6^ evaluated the effects of dual-cured resin cement, dual-cured resin cement plus polyethylene fiber, and dual-cured resin cement plus glass fiber on fracture resistance of roots with reattached fragments. According to the results, separated fragments of vertically fractured teeth can be reattached by using a dual-cured resin or by adding polyethylene fiber. In the present study, Super-Bond C&B was compared with self-adhesive dual-cured resin cement. According to the results of the present study, the fracture resistance values of self-adhesive dual-cured resin cement group were lower than those of the control and Super-Bond C&B groups. Discrepancies in the results of studies could be explained by differences in methodologies and control groups.

In this study, single-rooted human teeth with similar dimensions were used. All the specimens were assigned to the groups in terms of the mesiodistal and buccolingual lengths. The root length was standardized as 15 mm in all the groups. The root canals were prepared and filled with the same techniques, and the other procedures were similar in all the groups. Although the controllable factors were standardized as much as possible, the standard deviations were relatively high, which might be due to the uncontrollable anatomic variations.

## Conclusion

Within the limitations of the present study, Super-Bond C&B was useful in obtaining higher fracture resistance in endodontically treated teeth with vertical root fractures.

## Authors’ Contributions

EDY & HA: Planning, Materials and methods stage, writing stage. NA: Materials and methods stage. MG: Materials and methods stage. AÖ Materials and methods stage. ECG: Materials and methods stage.

## Acknowledgements

None.

## Conflict of interests

The authors declare that they have no conflict of interests.

## Ethical approval

Not applicable.

## Funding

Not applicable.
